# Biocontrol fungi induced stem-base rot disease resistance of *Morinda officinalis* How revealed by transcriptome analysis

**DOI:** 10.3389/fmicb.2023.1257437

**Published:** 2023-12-01

**Authors:** Zien Chen, Panpan Han, Xiaoying Che, Zhenhua Luo, Zeyu Chen, Jinfang Chen, Tijiang Shan, Ping Ding

**Affiliations:** ^1^College of Traditional Chinese Medicine, Guangzhou University of Chinese Medicine, Guangzhou, China; ^2^College of Forestry and Landscape Architecture, South China Agricultural University, Guangzhou, China

**Keywords:** *Morinda officinalis* How, stem-base rot disease, *Fusarium oxysporum*, biological control, induced resistance, transcriptome analysis

## Abstract

**Introduction:**

*Morinda officinalis* How (MO) is a Rubiaceae plant, and its medicinal part is dried root, which is one of the “Four Southern Medicines” in China. At present, the plant MO breed seedlings mainly by cutting methods. Long-term asexual propagation makes pathogenic fungi accumulate in MO, leading to stem-base rot, which is caused by *Fusarium oxysporum* (Fon).

**Methods:**

In this study, we used *Trichoderma harzianum* and *Pestalotiopsis* sp. as biocontrol fungi to investigate their antagonistic ability to Fon through in vitro antagonism and pot experiments, and combined with transcriptome sequencing to explore the mechanism of biocontrol.

**Results:**

The results showed that both *Trichoderma harzianum* and *Pestalotiopsis* sp. could inhibit the growth of Fon. In addition, *Trichoderma harzianum* and *Pestalotiopsis* sp. could also enhance the basic immunity to Fon by increasing the activities of defensive enzymes such as POD and SOD, chlorophyll content, soluble sugar content, and oligosaccharide content of MO. The mechanism of biological control of stem-base rot of MO was discussed by transcriptome technology. MO was treated with two treatments, root irrigation with biocontrol fungi or inoculation with Fon after root irrigation with biocontrol fungi. Transcriptome sequencing revealed that nearly 11,188 differentially expressed genes (DEGs) were involved in the process of inducing MO systemic resistance to Fon by biocontrol fungi. Meanwhile, Gene Ontology (GO) classification and Kyoto Encyclopedia of Genes and Genomes (KEGG) pathway enrichment, as well as transcription factor (TFs) prediction showed that there were significant differences in the expression levels of MO roots under different treatments. Also, the genes of the “MAPK signaling pathway” and “plant hormone signaling pathway” were analyzed, in which the ERFs gene of the ethylene signal transduction pathway participated in the metabolism of glycosyl compounds. It is speculated that the ethylene signal may participate in the immune response of the sugar signal to the infection of Fon. After qRT-PCR verification of 10 DEGs related to the ethylene signal transduction pathway, the expression trend is consistent with the results of transcriptome sequencing, which proves the reliability of transcriptome sequencing.

**Discussion:**

In conclusion, this study preliminarily identified the molecular mechanism of the biological control of MO stem-base rot and provided a scientific basis for further research on the prevention and control mechanism of MO stem-base rot.

## 1 Introduction

*Morinda officinalis* How (MO) is a plant of the Rubiaceae family, with the medicinal part of dried root, which has the effects of tonifying kidney yang, strengthening bones and muscles, and expelling wind and dampness (National Pharmacopoeia Committee, 2020). It is one of the famous “Four Southern Herbal Medicines” in China. MO is mainly produced in Guangdong, Fujian, Guangxi, Hainan, and other provinces in China, among which Deqing County of Guangdong is an authentic producing area (Cai et al., 2021). According to morphology, MO can be divided into large leaf species, middle leaf species, small leaf species, and wild species, etc. (Zhang et al., 2016). From the previous study of our research group, it was found that the small leaf species MO had stronger resistance to stem-base rot. At present, MO seedlings are mainly propagated by cutting, and long-term asexual propagation leads to the accumulation of pathogenic fungi. And MO is a perennial plant, with the extension of planting years, stem-base rot often occurs. MO stem-base rot is also known as MO *Fusarium* wilt, and the pathogenic fungus is *Fusarium oxysporum* (Fon) (Orr and Nelson, 2018). The disease is commonly seen in wheat, corn, and other crops, and a few in medicinal plants such as atractylodes and codonopsis (Jevtić et al., 2019; Qi et al., 2021; Shen et al., 2021; Wang et al., 2022). The symptoms of the disease first appeared in the stem base. In the early stage, the cortex of the stem base showed reddish-brown spots, which expanded into severe disease spots in the late stage, showing water stains. Subsequently, the vascular bundle became violet-brown, and the cortex of the stem base began to rot and deteriorate, with white mycelium visible (McNally et al., 2018; Castillo et al., 2022). The disease can cause serious quality damage and yield loss during MO production. Therefore, it is valuable and urgent to find effective and environmentally friendly methods to reduce the damage caused by Fon to MO production.

Biological control mainly uses antagonistic fungi, bacteria, actinomyces, and other antagonistic bacteria as well as plant-derived active substances, biocontrol microorganisms, or mixed with organic fertilizers. Because of its environment-friendly characteristics, it has a good application prospect in agriculture (Sharma, 2023). The biological control of stem-base rot is mainly through the antagonism of some microorganisms to induce systemic disease resistance in plants (Zhou et al., 2021). At present, more antagonistic microorganisms have been studied, including *Streptomyces*, *Bacillus*, and *Trichoderma harzianum* (Dor et al., 2007). *Trichoderma harzianum* is a common biological control fungus, which can inhibit the growth of pathogenic fungi through heavy parasitism and its active components also have antagonistic effects, even better than chemical reagents (Yassin et al., 2021). In addition, Kumar et al. (2021) found that *Trichoderma* sp. can activate antioxidant mechanisms in plants, such as peroxidase, polyphenol oxidase, superoxide dismutase, catalase, and ascorbic acid. Moreover, the potential ability of *Trichoderma* sp. as a biocontrol agent against various plant diseases has also been studied (Asad, 2022). In addition to the biocontrol fungi listed above, many other species can be used as biocontrol agents to control stem-base rot. Most species of *Pestalotiopsis* sp. have been studied as plant pathogenic fungi for more than a century, until the 1990s, when endophytic *Pestalotiopsis* sp. was first reported (Steyaert, 1953; Mordue, 1985; Strobel et al., 1997; Photita et al., 2001). However, whether endophytic *Pestalotiopsis* sp. can antagonize the pathogen has not been reported, and it can be further studied.

The effects of biocontrol fungi on pathogens are usually multiple inhibition mechanisms, which are coordinated and integrated to achieve the purpose. Biocontrol fungi can be divided into two mechanisms, including direct inhibition mechanism and indirect inhibition mechanism. The direct mechanism can be divided into hyperparasitism, competition, antagonism, and lysis, the indirect mechanisms include induction and growth promotion. In the common microecological environment, biocontrol fungi compete with pathogens for limited space and nutrition resources through rapid propagation and growth, which is called competition. Sa et al. (2021) found that *Bacillus subtilis* N6-34 could colonize rhizosphere soil and poplar plants and inhibit the growth of pathogens. In addition, biocontrol fungi directly or indirectly inhibit the growth of pathogens by secreting metabolites and antibacterial substances. Swain et al. (2008) studied the interaction between *Bacillus subtilis* CM1 and CM3 and Fon by scanning electron microscopy and found that the chitin and β-1, 3-glucanase produced by CM1 and CM3 distorted the mycelium of Fon. The important defense mechanisms of plant resistance mediated by microorganisms include systemic acquired resistance (SAR) and induced systemic resistance (ISR) (Narware et al., 2023). Many effective biocontrol fungi can induce ISR, and some related research results have been reported in recent years. For example, the *Pseudomonas fluorescen*t WCS417r-mediated ISR antagonize various plant pathogens in Arabidopsis by activating the JA signaling pathway (Pieterse et al., 1998). Recent studies have shown that ISR is also one of the important mechanisms of biological control of Fon. According to the report, *Trichoderma gamsii* can induce ISR against Fon by different pathways (Stefania et al., 2020). Therefore, further analysis of ISR induced by biocontrol fungi is of great significance for the biological control of stem-base rot.

At present, chemical control is mainly used to control stem-base rot in MO, which can easily cause environmental pollution. In this study, we identified two efficient biocontrol fungi *Trichoderma harzianum* and *Pestalotiopsis* sp. which can be used for Fon control in MO. The results showed that biocontrol fungi could inhibit the growth of Fon *in vitro*. In addition, biocontrol fungi can enhance the immunity of plants to Fon by raising the activity of defensive enzymes, the content of chlorophyll, and the content of soluble sugar, and also affect the content of MO oligosaccharide. To explore the control mechanism of biocontrol fungi, transcriptome sequencing was used for analysis. A total of 921,532 unigenes were identified in MO, and transcriptome analysis showed that there were more than 10,000 DEGs in biocontrol fungi against Fon. These genes were annotated and enriched for GO functions and KEGG pathways, as well as predicted TFs, and validated using real-time PCR (qRT-PCR). The results showed that the expression levels of MO roots were significantly different after different biocontrol fungi treatments. We also analyzed the MAPK signaling pathway and plant hormone signaling pathway and related genes involved in the control process. In conclusion, this study aims to understand the mechanism of systemic resistance of MO to Fon induced by biocontrol fungi from the molecular level, providing a scientific basis for further analysis of the biocontrol mechanism.

## 2 Materials and methods

### 2.1 Plant material, fungi, and growth condition

*Morinda officinalis* used in this experiment came from Deqing County, Guangdong, China, and was detoxified by tissue culture. When the tissue culture seedlings were rooted and transplanted into sterile nutrient soil for 6 months.

*Fusarium oxysporum* (ON454550) and *Trichoderma harzianum* (ON454551) were isolated from the root of *Morinda officinalis* and identified and preserved by our research group. And a strain of *Pestalotiopsis* sp. (OR608750)with antibacterial activity proposed by the College of Forestry and Landscape Architecture, South China Agricultural University. The pathogens and biocontrol fungi were cultured on potato dextrose agar (PDA) medium at 25°C under dark conditions for 5 days. The microconidia suspension of the pathogens and biocontrol fungi used in greenhouse experiments was prepared as described by Sabry et al. (2022).

### 2.2 Effects of biocontrol fungi on fungal antagonism and hyphal growth

To evaluate the biocontrol potential of *Trichoderma harzianum* and *Pestalotiopsis* sp., we evaluated the effect of fungal antagonism and hyphal growth. For the fungal antagonism, we used the flat antagonism experiment on the PDA medium *in vitro*. The six-millimeter plugs from 5-day-old cultures of the pathogens and biocontrol fungi were inoculated on both ends of the PDA culture medium plate, and the distance between biocontrol fungi and pathogens was 5 cm. After incubation at 28°C for 7 days, the colonies’ diameters of pathogens were determined. The experiment was repeated three times with only pathogenic bacteria inoculated as control. The inhibition rate was calculated according to the following formula: inhibition rate (%) = [(colonies’ diameter of the control group–colonies’ diameter of treatment group)/colonies’ diameter of control group] × 100% (Zhang et al., 2021).

The effect of biocontrol fungi on the hyphal growth of pathogens was detected according to the method of Kong et al. (2022) with minor modifications. Spreading a 1 mm thick PDA culture medium on a sterile glass slide, the six-millimeter plugs from 5-day-old cultures of the pathogens and biocontrol fungi were inoculated on both ends of the glass slide, 2 cm apart. Each treatment was repeated 3 times, cultured at 28°C for 24 ~ 36 h, observed by a microscope (Olympus Microscope BX43F), and photographed after biocontrol fungi contacted with Fon hyphae.

### 2.3 Experimental design in greenhouse

To evaluate the biocontrol effect of biocontrol fungi on MO stem-base rot, a greenhouse experiment was carried out in this study. Six months after transplanting tissue culture seedlings of MO into sterile nutrient tanks, plants were treated with 100 mL single biocontrol fungus or combined biocontrol fungi (1 × 10^6^ conidia /ml, T, P, TP), and sterile water was used as a mock control (NF). After 24 h, each treated plant was inoculated with 100 mL Fon spore suspension (1 × 10^6^ conidia/ml, FT, FP, FTP), and Fon alone was used as a control (F). Disease index (DI) was recorded and photographed 30 days after inoculation, and the roots of MO were harvested for RNA sequencing and determination of oligosaccharide content. In addition, the leaves of MO at different time points were harvested for the determination of physiological and biochemical indexes.

### 2.4 Determination of physiological and biochemical indexes and oligosaccharide content

At harvest, we analyzed the activity of plant defense-related enzymes (SOD and POD), chlorophyll content, and soluble sugars during the process of biocontrol fungi triggering ISR to stem-base rot in MO. The above physiological and biochemical indicators were determined by using the corresponding detection kit, namely “The SOD detection kit” and “The POD detection kit” (Nanjing Jiancheng Biological Engineering Institute, Nanjing, China), “The Chlorophyll content test kit” (Leagene, Beijing, China) and “The Soluble sugar content detection kit” (Solarbio, Beijing, China) according to the manufacturer’s instructions. The oligosaccharide content was determined according to the method of Liu et al. (2022).

### 2.5 RNA extraction, library construction, and RNA sequencing

Total RNA was extracted by Total RNA Extractor (Trizol) (Sangon Biotech, Shanghai, China) according to the manufacturer’s instructions. The concentration of RNA was measured by Qubit2.0 (Thermo Fisher Scientific, United States), and the integrity of RNA and genomic contamination were detected by agarose gel.

A total of 24 samples were used for library construction in this study. Of these, 12 samples were obtained from MO treated with *Trichoderma harzianum* or *Pestalotiopsis* sp. alone (T, P), *Trichoderma harzianum* mixed with *Pestalotiopsis* sp. (TP), and sterile water treatment (NF). The other 12 samples were pretreated with the above biocontrol fungi for 24 h and then challenged with Fon (FT, FP, FTP, F). All samples were taken from at least 5 MO roots and stored in a −80°C refrigerator. The total RNA of each sample was isolated by the above method.

The library was constructed using the qualified total RNA above. The first-strand cDNA was synthesized by the 1st Strand Synthesis Reaction Buffer (Sangon Biotech, Shanghai, China), and the second cDNA strand was synthesized immediately after the reaction. After the terminal was repaired and the sequencing joint was connected, the junction product was purified and fragment size was sorted by magnetic beads. Finally, PCR amplification was performed with 2 × Super Canace™ High-Fidelity Mix and Primer mix reagent (YEASEN, Shanghai, China). Library amplification products were purified using Hieff NGS™ DNA Selection Beads (0.9×, Beads: DNA = 1:1) (YEASEN, Shanghai, China). The library was detected by agarose gel electrophoresis, and the recovered DNA was accurately quantified by a Qubit DNA detection kit (YEASEN, Shanghai, China). Illumina sequencing was carried out in the Shanghai Sangon Biotech, China, and performed using the Illumina MiSeq™ platform according to the manufacturer’s instructions (Illumina, United States).

### 2.6 *De novo* assembly, functional annotation, and differentially expressed genes analysis

The raw image data files obtained from Illumina Miseq™ were converted into raw sequenced reads by CASAVA Base Calling analysis, which contained the sequence information of the reads and the corresponding sequencing quality information. The raw data quality values and other information were counted, and the sequencing data quality of the samples was evaluated visually by using FastQC. Raw data from sequencing, which contains low-quality sequences with junctions. To ensure the quality of information analysis, the raw data must be filtered to obtain clean data. In this study, clean data *de novo* was assembled into transcripts using Trinity, and sequencing data were assembled into transcripts through three steps: Inchworm, Chrysalis, and Butterfly.

The transcript obtained by Trinity assembly was deredundant, and the longest transcript in each transcript cluster was taken as Unigene, which was used as the reference sequence for subsequent analysis. The Unigene was compared with CDD, KOG, COG, NR, NT, PFAM, and GO databases using Blast alignment software, and the KEGG database was compared using KAAS alignment software to obtain the annotation information of Unigene.

All Unigene was compared with the reference sequence, and the comparison results were calculated by RSeQC. Then, according to the comparison results and the annotation file of the reference genome, the read count data was standardized by TMM, and then the difference analysis was conducted by DEGseq. In order to get a significant difference of gene will filter is set to: qValue <0.05, and the difference between multiples | FoldChange | > 2. Then all DEGs were subjected to GO and KEGG functional enrichment analysis, and when the corrected *p*-value (Qvalue) < 0.05, the function was considered to be significantly enriched. In addition, to identify all transcription factors (TFs) in the root of MO, all Unigene was compared to the plant transcription factor database for TFs prediction.

### 2.7 Validation of DEGs by qRT-PCR

To confirm the transcriptome data, 10 DEGs related to the ethylene signaling pathway were randomly selected and verified (Supplementary Table S1). The total RNA of the sample was reverse transcribed using the Color Reverse Transcription Kit (with gDNA Remover) (EZBioscience, Beijing, China). qRT-PCR was conducted on Qtower3 Real-Time PCR Instrument (Jena, Germany) by using the 2 × Color SYBR qPCR Master Mix (ROX2 puls) (EZBioscience, Beijing, China). The reaction was performed under the following conditions: 95°C for 8 min, followed by 40 cycles of 95°C for 10 s, and 60°C for 30 s. The actin gene of MO was employed as the internal standard. All the gene information and PCR primers used in our study are shown in Supplementary Table S1.

### 2.8 Statistical analysis

All bioassays and experiments were performed three times with 5 seedlings treated each time. Significant differences between means were compared using the LSD test (Fisher’s protected most significant difference test) at *P*≧ 0.05. *p*-value < 0.05 was considered statistically significant. All data were analyzed by analysis of variance using SPSS 20.0 (SPSS Inc., Chicago, IL).

## 3 Results

### 3.1 Antagonistic effect of biocontrol fungi *in vitro*

**Figure 1:**
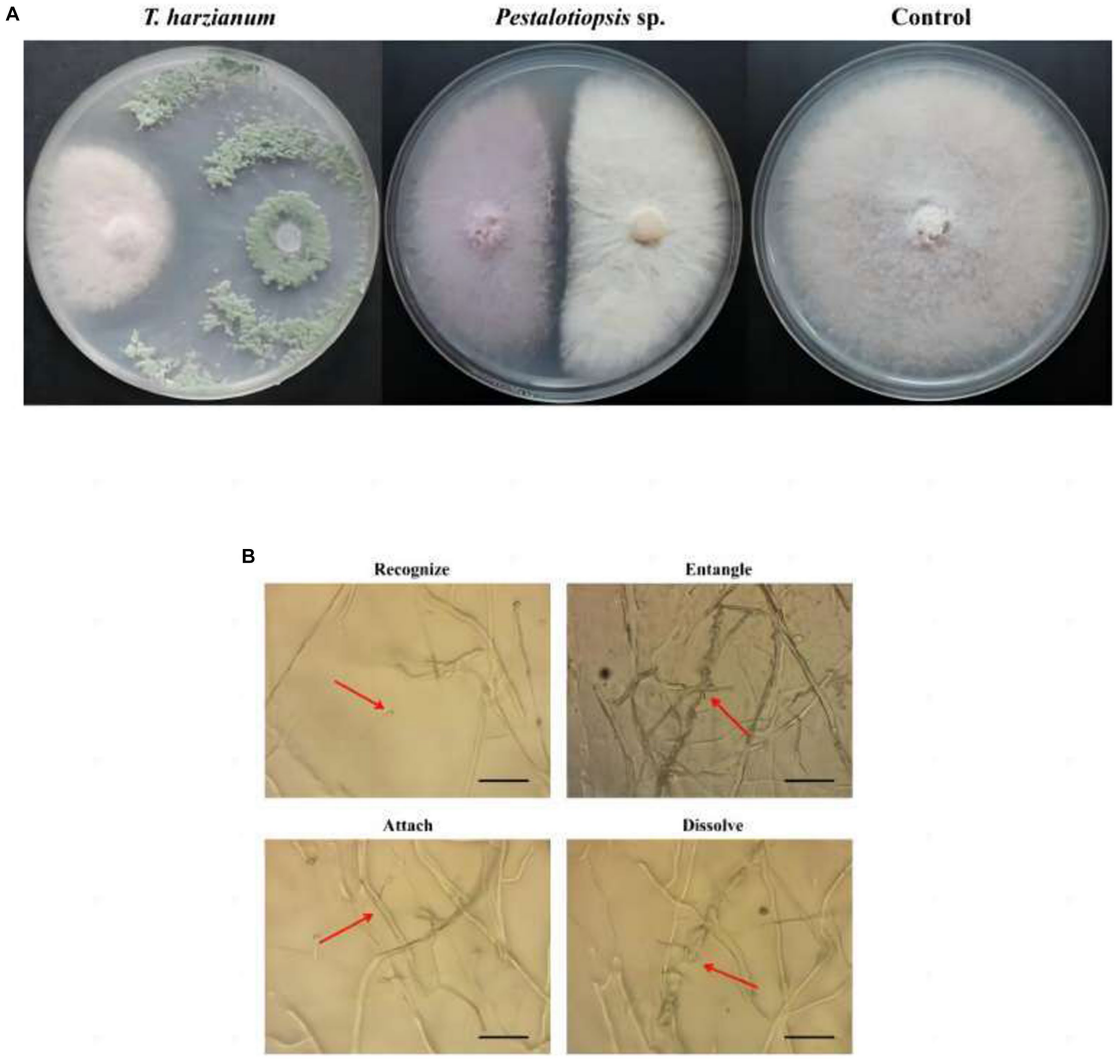
Effects of biocontrol fungi on fungal antagonism and parasitism. **(A)** Antagonistic effect of biocontrol bacteria on Fon. **(B)** The parasitic effect of biocontrol fungi on Fon was observed under the microscope (40×).

Through antagonistic experiments, the results showed that *Trichoderma harzianum* and *Pestalotiopsis* sp. have antagonistic activity against Fon *in vitro* (Figure 1A). The inhibition rate of *Trichoderma harzianum* against Fon was 58.30% (*p* = 0.000), and the inhibition rate of *Pestalotiopsis* sp. against Fon was 33.81% (*p* = 0.000) (Supplementary Table S2). Through microscope observation, it was found that in the process of antagonizing Fon, the biocontrol fungi first recognized Fon, then parasitically parasitized on Fon mycelia by way of entanglement or attachment, and finally penetrated and dissolved Fon mycelia (Figure 1B).

### 3.2 Effect of biocontrol fungi in greenhouse experiment

**Figure 2:**
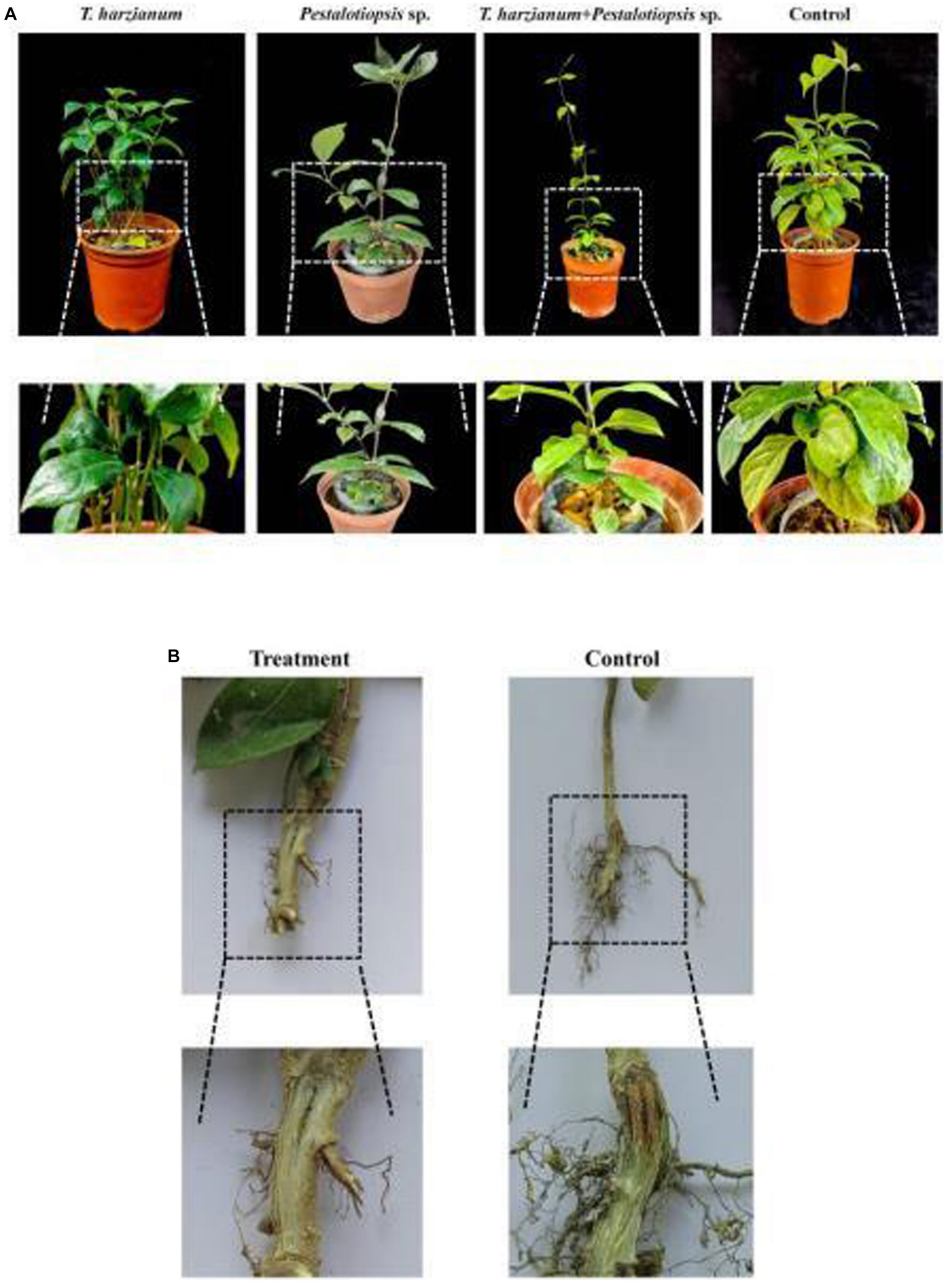
Greenhouse experiment with different bacterial treatments. **(A)** Symptoms of stem-base rot in different treatments of MO plants 30d after inoculation with Fon in a greenhouse experiment. **(B)** Degree of vascular browning in the base-stem of MO after biocontrol fungi treatment.

Based on the above results, we found that *Trichoderma harzianum* and *Pestalotiopsis* sp. have the potential to control MO stem base rot. To test this hypothesis, we conducted a greenhouse experiment. After 30 days of inoculation with Fon, the disease symptoms of the control group were obvious, while the stem-base rot symptoms of MO treated with biocontrol fungi were significantly reduced. Among them, the combined treatment of *Trichoderma harzianum* and *Pestalotiopsis* sp. had the most obvious effect, with a control effect of 46.67% (*p* = 0.000), while the control effect of biocontrol fungus alone was 36.36% (*p* = 0.010) (Figure 2A; Supplementary Table S3). In addition, we also found that the degree of vascular browning after biocontrol fungi treatment was significantly lower than that in the control group, indicating that biocontrol fungi could effectively delay the infection process of Fon (Figure 2B).

### 3.3 Determination of physiological and biochemical indexes and oligosaccharide content

**Figure 3:**
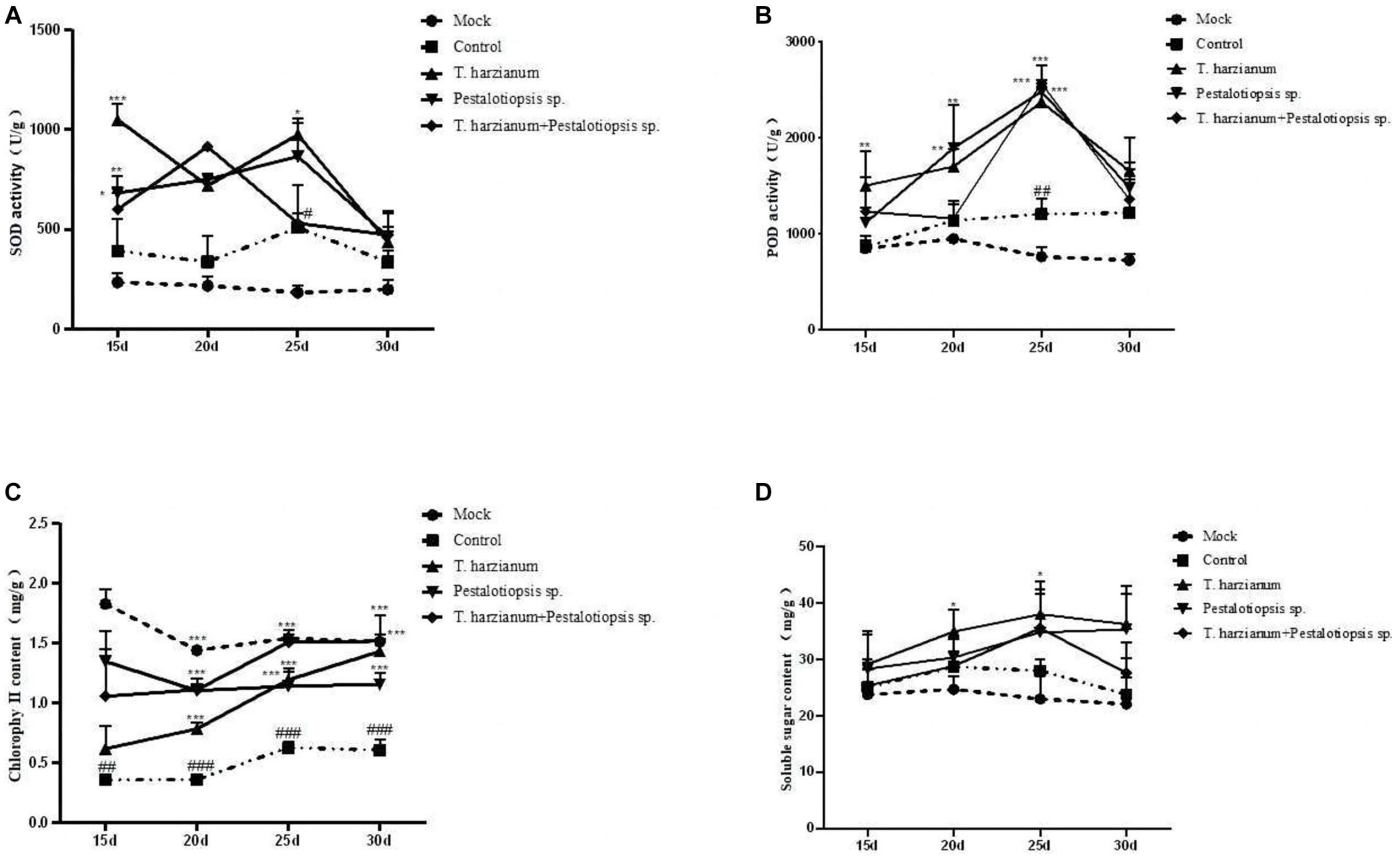
Effects of biocontrol fungi on physiological and biochemical indexes of MO which was pretreated with biocontrol fungi and challenged with Fon. **(A,B)** The activities of defense-related enzymes (SOD and POD) in the leaves of MO. **(C)** The content of chlorophyll in the leaves of MO. **(D)** The content of soluble sugar in the leaves of MO.

The SOD activity and POD activity after each biocontrol treatment were significantly higher than that of the control group and showed a trend of first increasing and then decreasing as time went by (Figures 3A,B). These results indicated that the disease resistance of Mo was enhanced by using biocontrol fungi. The chlorophyll content of MO after biocontrol treatment was higher than that of the control group, but lower than that of the normal group (Figure 3C). With the change of time, except for *the Trichoderma harzianum* treatment group, the total chlorophyll content of other biocontrol fungi treatments had little change, while the total chlorophyll content of *the Trichoderma harzianum* treatment group would increase as time went by. The soluble sugar content of the biocontrol treatment was higher than that of the control group and showed a trend of increasing first and then decreasing as time went by Figure 3D. Compared with the normal group, the oligosaccharide content in the control group increased. Compared with the control group, the contents of 1-ketose and nystose in each biocontrol treatment group were decreased, while the contents of fructose and glucose were increased, which was consistent with the results of soluble sugar content (Supplementary Table S4).

### 3.4 Transcriptome sequence and assembly

The quality and concentration of all RNA samples were tested by Qubit2.0. The RNA quality of all samples was high, with an OD value above 2.0. The samples with unqualified concentration and quality were re-extracted to ensure that all samples met the requirements of the transcriptome sequencing library.

**Figure 4:**
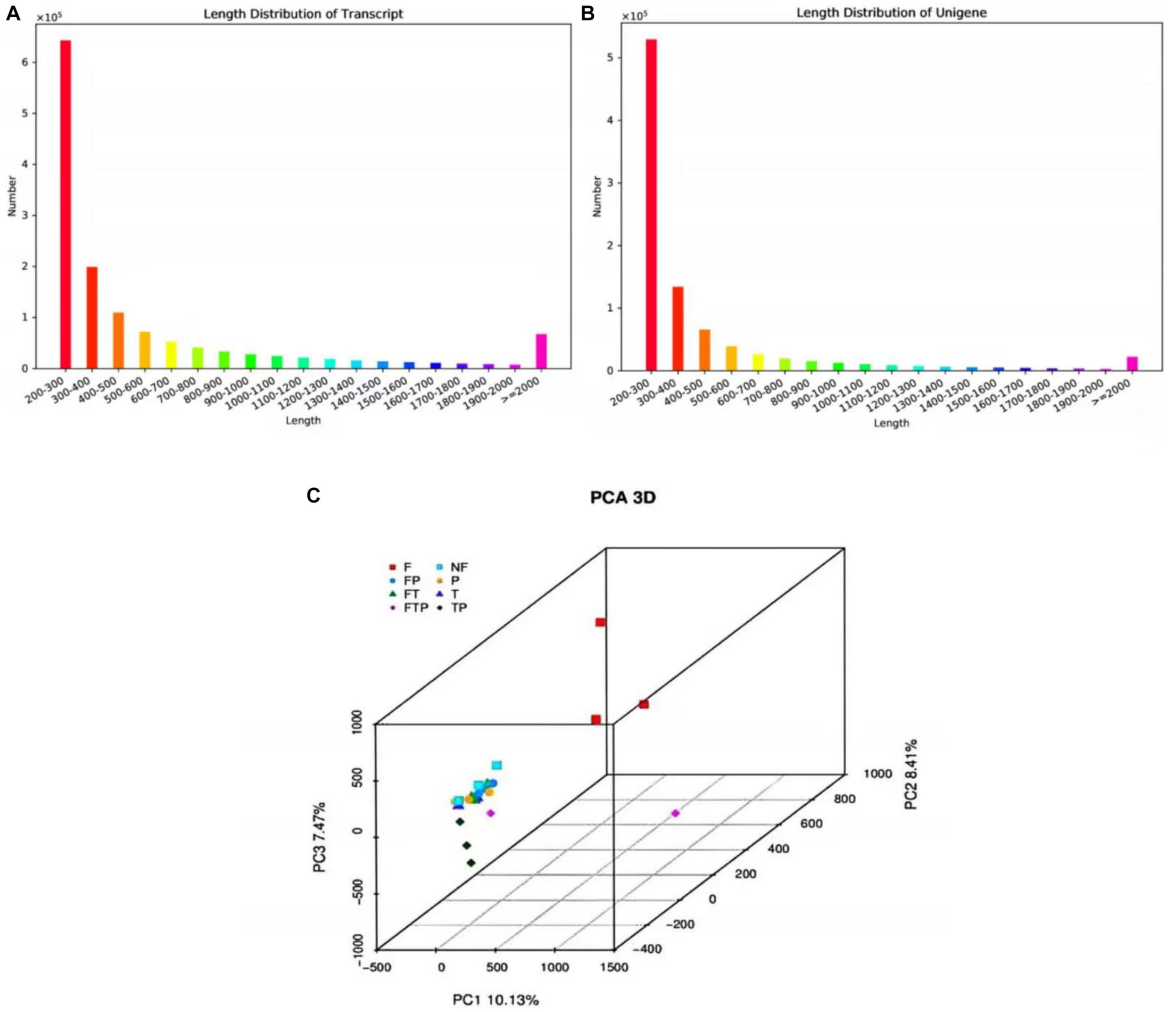
The sequence length distribution plot and principal component analysis. **(A)** Transcript sequence length cumulative distribution. **(B)** Unigene sequence length cumulative distribution. The horizontal axis is the length interval, and the vertical axis is the number of sequences in the interval. **(C)** Principal component analysis.

A total of 583,671 million raw reads were obtained by high-throughput sequencing, and filtering of the raw data resulted in 522,375 million clean reads, representing 90% of the raw reads. The Q20 bases ranged from 97.79 to 98.46%, Q30 ranged from 92.82 to 94.68, and the GC amount ranged from 46.36 to 57.52%, indicating that the sequencing accuracy is high and can meet the requirements of subsequent analysis (Supplementary Table S5). The 1,390,679 transcripts obtained from the trinity assembly yielded 921,532 unigenes with an average length of 461.48 bp and N50 of 547 bp, and the length distribution of these unigenes is shown in Figures 4A,B. The longest transcript in each transcript cluster was used as the reference sequence for subsequent analyses. From the principal component analysis (PCA) it can be seen that the distribution of the samples in each group is more clustered, indicating that there is less variation within the samples, whereas analyzing the variation between groups shows that the control group is more dispersed from the rest of the component samples with significant differences (Figure 4C).

The similarity of Unigenes sequences was compared with seven public databases (CDD, KOG, COG, NR, NT, PFAM, and GO) using blast (E-value≤10^−5^). The unigenes sequences were aligned to the KEGG database using the KAAS software. A total of 921,532 Unigenes were obtained annotated, of these, 176,943 (19.2%) were annotated to the CDD database, 204,656 (22.21%) were annotated to the PFAM database, 191,958 (20.83%) were annotated to the KEGG database, 228,126 (24.76%) were annotated to the KOG database, 268,497 (29.14%) were annotated to the GO database, 417,346 (45.29%) were annotated to the NR database, 47,654 (5.17%) were annotated in the NT database (Supplementary Table S6).

### 3.5 DEGs analysis and functional annotation of different biocontrol fungi treatments

**Figure 5:**
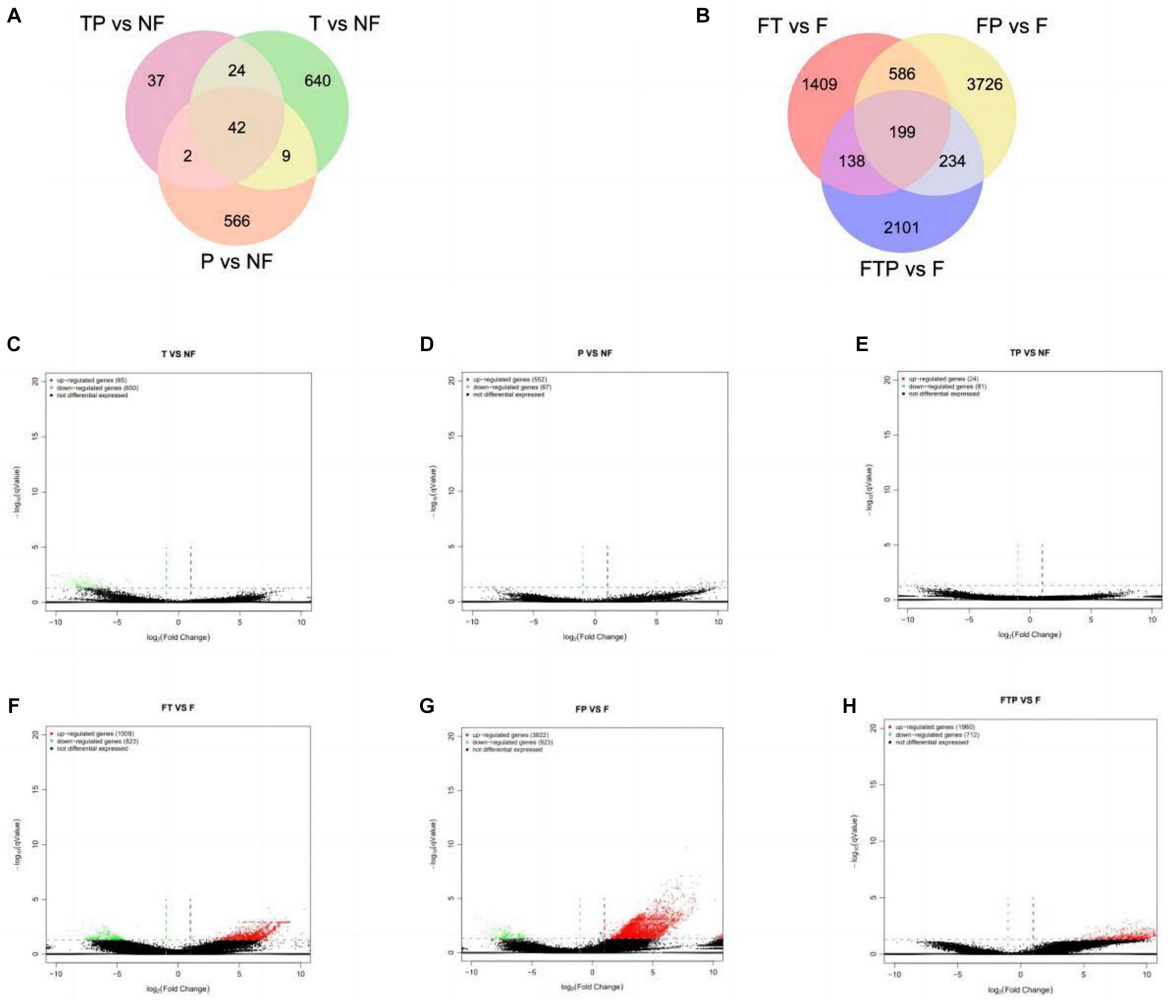
Classification of differentially expressed genes (DEGS) in the root of MO in response to different biocontrol fungi treatment or in combination with Fon inoculation. **(A)** Venn diagram of DEGs identified in the root of MO treated with biocontrol fungi alone. **(B)** Venn diagram of DEGs identified in the root of MO pretreated with biocontrol fungi and challenged with Fon. **(C–E)** Volcanic map of DEGs identified in the root of MO treated with biocontrol fungi alone. **(F–H)** Volcanic map of DEGs identified in the root of MO pretreated with biocontrol fungi and challenged with Fon. The horizontal axis is the fold-change (log (B/A)) value of gene expression difference between different groups, and the vertical axis is the pValue of the statistical significance of gene expression change. The smaller the pValue and the larger the −log (pValue), the more significant the difference. Red indicates up-regulated genes, green indicates down-regulated genes, and black indicates non-differential bases.

Based on the above sequencing data, two control groups were established to study the control mechanism of different biocontrol bacteria on stem-base rot of MO. In one group, only treated with biocontrol fungi and compared with the sample treated with sterile water. The other group was inoculated with the pathogen Fon after being pretreated with biocontrol fungi for 24 h and compared with the sample inoculated with Fon after sterile water treatment. It was observed that the expression levels of a large number of genes in the root of MO were changed whether the biocontrol fungi were treated alone or pretreated before inoculation with pathogenic bacteria (Figures 5A,B). According to the qValue <0.05 multiples and differences | FoldChange | > 2 conditions of screening DEGs analysis, according to the results by the Figures 5C–H, in the group of only treated with biocontrol fungi, 715 DEGs (65 up-regulated genes, 650 down-regulated genes) were detected in the *Trichoderma harzianum* treatment group, 619 DEGs (552 up-regulated genes, 67 down-regulated genes) were detected in the *Pestalotiopsis* sp. treatment group, and 105 DEGs (24 up-regulated genes, 81 genes downregulated) were detected in the co-treatment group with *Trichoderma harzianum* and *Pestalotiopsis* sp. In the samples pretreated with biocontrol bacteria, 2,332 DEGs (1,509 up-regulated genes, 823 down-regulated genes) were detected in the *Trichoderma harziamum* group, and 4,745 DEGs (3,822 up-regulated genes, 923 down-regulated genes) were detected in the *Pestalotiopsis* sp. group. A total of 2,672 DEGs (1960 up-regulated genes, 712 down-regulated genes) were detected in the co-treatment group of *Trichoderma harzianum* and *Pestalotiopsis* sp.

The selected DEGs were compared with the GO database for functional annotation. The GO database was divided into 3 categories, including “biological process,” “cellular component” and “molecular function,” which were further divided into 68 sub-categories. The results showed that compared with mock treatment or Fon inoculation alone, the most DEGs were annotated to the “cellular process,” “metabolic process” and “cell component organization or biogenesis” in the category of “biological process” in the libraries constructed from MO treated with biocontrol fungi alone or pretreated with biocontrol fungi and then inoculated with Fon. In the category of “cell component,” the number of DEGs annotated as “cell components,” “cells” and “organelles” was the most, while the number of DEGs annotated as “binding” and “catalytic activity” in the category of “molecular functions” was the most (Supplementary Figure S1). These results indicate that the physiological and biochemical functions of MO can be changed by biocontrol bacteria in the process of stem-base rot control.

The selected DEGs were compared with the KEGG database to analyze the metabolic pathways in MO. According to the results (Supplementary Figure S2), KEGG pathways are divided into five categories, namely “cell process,” “environmental information processing,” “genetic information processing,” “metabolic pathway” and “organic system.” The five categories could be further divided into 33 sub-categories, which were mainly annotated in the “transport and catabolism,” “signal transduction,” “translation,” “folding and explanation,” “carbohydrate metabolism,” and “energy metabolism” pathway. The above analysis of the KEGG metabolic pathway may lay a foundation for the study of the biological control mechanism of MO stem-base rot.

### 3.6 GO function and pathway enrichment analysis for DEGs

GO functional enrichment analysis of DEGs in the group treated with biocontrol fungi alone showed that the functions of DEGs enriched by different biocontrol treatments were also different (Supplementary Figure S3). When treated with *Trichoderma harzian*um alone, DEGs were obviously enriched in the “oxidation reduction process,” “antibiotic metabolism process” and “cell response to oxidative stress.” When treated with *Pestalotiopsis* sp. alone, DEGs were significantly enriched in the “endoplasmic reticulum subcompartment,” “epigenuclear membrane-endoplasmic reticulum network,” and “cytoplasmic stress granules.” After *Trichoderma harzian*um and *Pestalotiopsis* sp. were treated together, only the “mitochondrial respiratory chain supercomplex” function of cell group classification was significantly enriched in DEGs, which may be related to the low amount of DEGs in this treatment.

**Figure 6:**
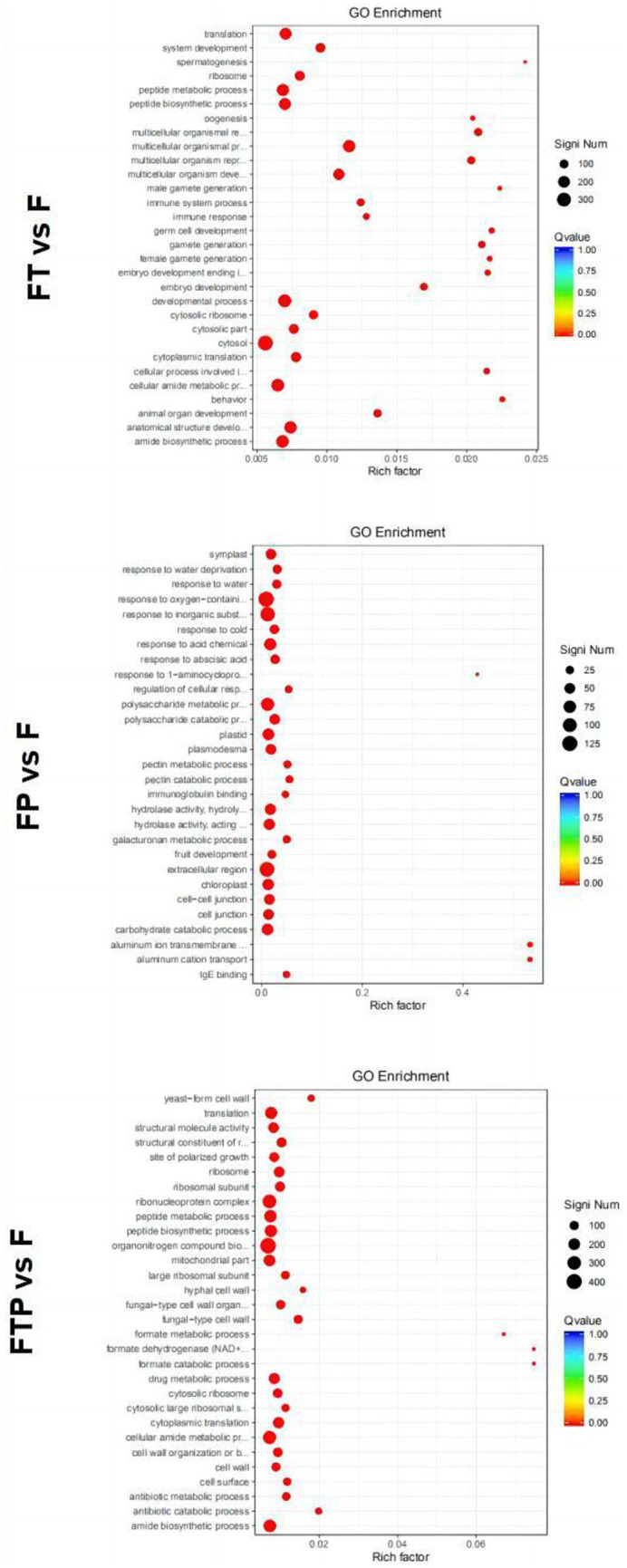
Significant enrichment GO function scatter diagram of DEGs. Significant enrichment GO function of DEGs in the comparisons of MO pretreated with biocontrol fungi and then inoculated with Fon. The vertical axis represents the functional annotation information, and the horizontal axis represents the Rich factor corresponding to the function (the number of differentially annotated genes for the function divided by the number of genes annotated for the function). The size of the Qvalue is represented by the color of the point, where the smaller the Qvalue, the redder the color. The number of differential genes included under each function is represented by the size of the dots. (Only select the top 30 GO with the highest enrichment degree to draw).

GO function enrichment analysis of DEGs in the biocontrol pretreatment group showed that DEGs after biocontrol pretreatment was significantly enriched in different functions (Figure 6). After pretreatment by *Trichoderma harzian*um, DEGs were significantly enriched in the “multicellular biological process,” “multicellular biological development,” “multicellular biological reproduction process,” “cytoplasmic ribosome,” “ribosome” and “molecular structural activity.” After pretreatment by *Pestalotiopsis* sp., DEGs were significantly enriched in “polysaccharide catabolic process,” “reaction to acidic chemicals,” “reaction to inorganic substances,” “extracellular region,” “cytoplasmic desmata,” “hydrolase activity, hydrolyzed O-linked glycosyl compounds”, “Hydrolase activity, acting on glycosyl bonds” function. After co-treatment with *Trichoderma harzian*um and *Pestalotiopsis* sp., DEGs significantly enriched “cytoplasmic translation,” “antibiotic catabolic process,” “organic nitrogen compound biosynthesis process,” “fungus-type cell wall,” “ribosome,” “structural components of ribosomes,” “formate dehydrogenase (NAD+) activity” and “structural molecular activity.”

By analyzing the enrichment of DEGs in the KEGG pathway of the group treated with biocontrol fungi alone, the results showed that the top 30 KEGG pathways of DEGs under different biocontrol bacteria treatments were different (Supplementary Figure S4). The DEGs of the group treated with *Trichoderma harzian*um alone could be enriched into the “MAPK signal pathway,” “P53 signal pathway,” “cGMP-PKG signal pathway,” “carbon metabolism,” etc. The DEGs of the group treated with *Pestalotiopsis* sp. alone could be enriched into “MAPK signal pathway,” “starch and sucrose metabolism,” “fatty acid metabolism,” etc. DEGs in the co-treatment group of *Trichoderma harzian*um and *Pestalotiopsis* sp. could be enriched to “MAPK signaling pathway,” “phenylpropane synthesis pathway,” “carbon metabolism” and “tyrosine metabolism.” All the above three treatments can enrich MAPK signaling pathways related to plant immunity and carbohydrate metabolism, indicating that the basic immunity of plants can be improved after biocontrol bacteria alone treatment.

**Figure 7:**
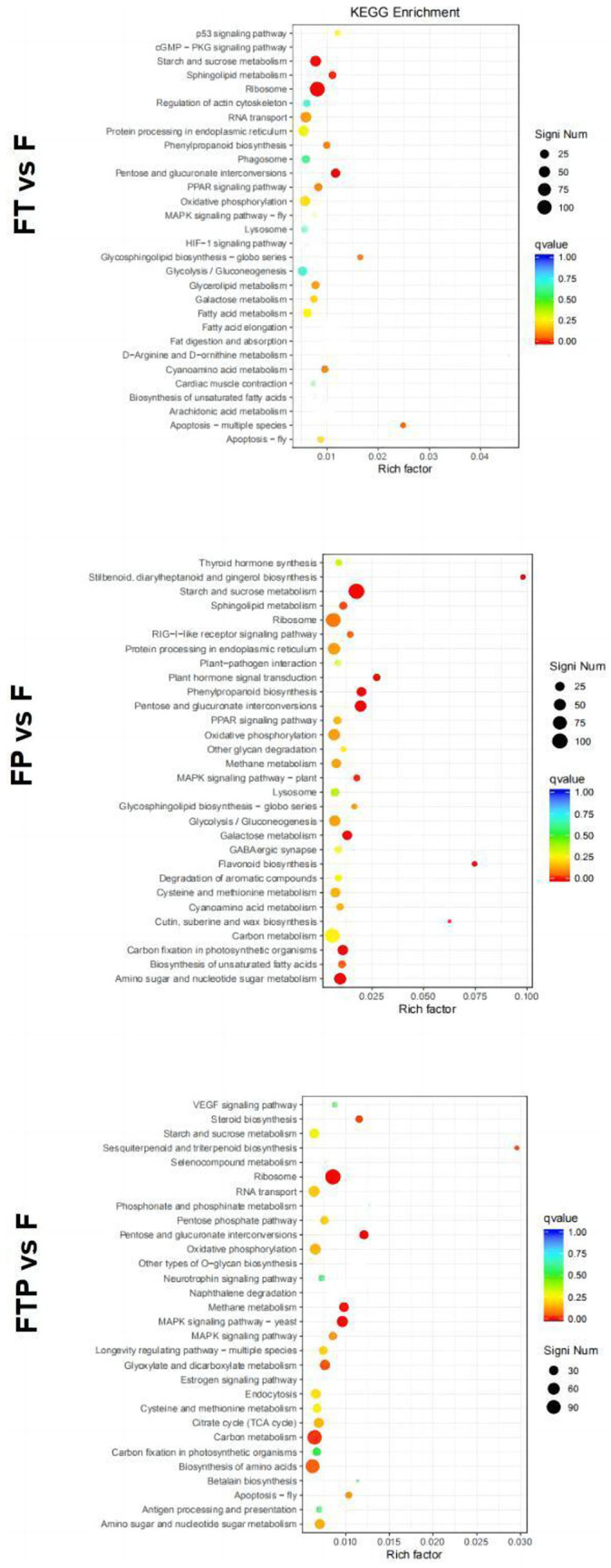
Significant enrichment KEGG pathway scatter diagram of DEGs. Significant enrichment KEGG pathway of DEGs in the comparisons of MO pretreated with biocontrol fungi and then inoculated with Fon. The vertical axis represents the functional annotation information, and the horizontal axis represents the Rich factor corresponding to the function (the number of differentially annotated genes for the function divided by the number of genes annotated for the function). The size of the Qvalue is represented by the color of the point, where the smaller the Qvalue, the redder the color. The number of differential genes included under each function is represented by the size of the dots. (Only select the top 30 KEGG with the highest enrichment degree to draw).

Enrichment in the KEGG pathway of DEGs pretreated with biocontrol fungi showed that the KEGG pathway related to plant immunity was significantly enriched in each group (Figure 7). In *the Trichoderma harzian*um pretreatment group, DEGs were significantly enriched in the “phenylpropane synthesis pathway” and “mutual conversion of pentose and glucuronic acid,” while in *the Pestalotiopsis* sp. pretreatment group, DEGs were significantly enriched in “MAPK signal pathway,” “phenylpropane synthesis pathway,” “plant hormone signal transduction” and “plant-pathogen interaction.” In the pretreatment group of *Trichoderma harzian*um and *Pestalotiopsis* sp., DEGs were significantly enriched in “MAPK signaling pathway,” “pentose and glucuronic acid interconversion” and “carbon metabolism.” In general, although the signal pathways of DEGs enrichment after pretreatment of different biocontrol bacteria are not consistent, all the above pathways can play an important role in the process of biological control.

### 3.7 Prediction of transcription factors during biocontrol fungi against Fon

**Figure 8:**
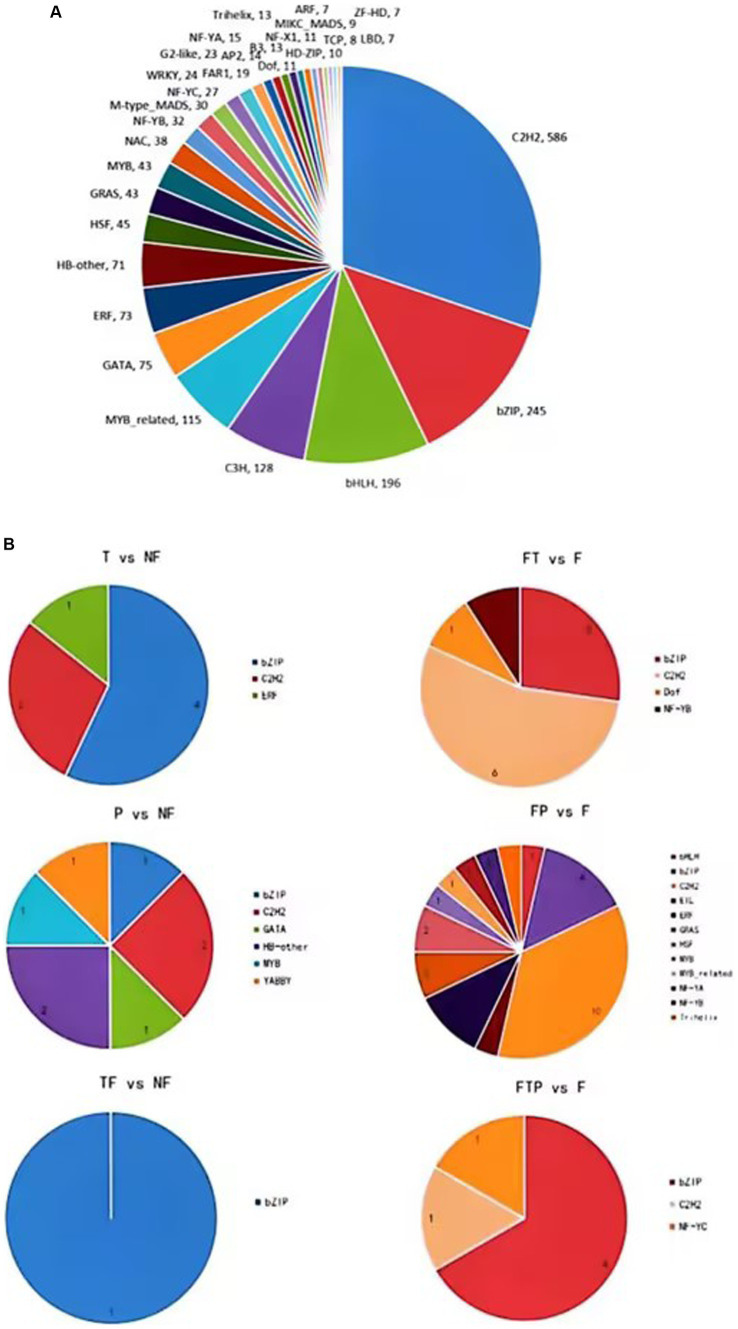
Transcription factors (TFs) prediction of DEGs. **(A)** Prediction of TFs in MO unigenes sequence. **(B)** Prediction of TFs of DEGs in biocontrol fungi treatment.

Transcription factors play an important role in plant growth and development, hormone signal transduction, leaf senescence, and plant disease resistance. In order to explore which transcription factors are related to the mechanism of biological control of stem-based rot, the transcriptome sequence of the root of MO was compared and analyzed with the plant transcription factor database (PlnTFDB2) (Figure 8). The results showed that a total of 2,017 genes were aligned to PlnTFDB2, and the aligned transcription factors could be divided into 53 categories, including transcription factors related to plant disease resistance, such as WRKY, MYB, bZIP, AP2, and NAC, and transcription factors related to plant hormone signal transduction, such as ARF, BZIP, ERF, and MYB transcription factors were differentially expressed significantly when the biocontrol agents were used alone, while bZIP, ERF, and C2H2 transcription factors were significantly differentially expressed after pretreatment with the biocontrol agents.

### 3.8 Gene expression level validation by qRT-PCR

To confirm the reliability of the RNA-seq data, 10 DEGs related to the ethylene signaling pathway were randomly selected for the qRT-PCR validation analysis. The results showed that after the biocontrol fungi were treated separately, the five DEGs of TRINITY_DN488910_c0_g1, TRINITY_DN529234_c1_g1, TRINITY_DN520737_c0_g2, TRINITY_DN520737_c0_g1, and TRINITY_DN505197_c1_g1 were all significantly down-regulated. TRINITY_DN537446_c0_g4 and TRINITY_DN533749_c2_g3 were up-regulated significantly, but the expression trend of some genes was inconsistent after different treatments. The TRINITY_DN532960_c2_g1 gene was up-regulated after *Trichoderma harzianum* alone treatment, but it was down-regulated significantly after the other treatments. The gene of TRINITY_DN519710_c1_g1 was down-regulated after co-treatment with *Trichoderma harzianum* and *Pestalotiopsis* sp., and all other treatments were significantly up-regulated, while the gene of TRINITY_DN517401_c2_g1 was up-regulated after treated with *Pestalotiopsis* sp. alone, and all the other treatments were significantly down-regulated (Supplementary Figure S5A). After biocontrol fungi pretreatment, the expression trends of the 10 DEGs in each group were consistent, and all the DEGs were significantly up-regulated after *Trichoderma harzianum* pretreatment, and down-regulated after *Pestalotiopsis* sp. pretreatment, but all the DEGs were up-regulated after the co-pretreatment with two biocontrol fungi (Supplementary Figure S5B). Although the qRT-PCR-verified DEGs expression levels were not consistent with transcriptome sequencing, the expression trends were consistent with transcriptome sequencing. These differences can be due to variable shearing, sample handling, and data analysis. There may be multiple transcripts in a gene, and the differential trend of each transcript may be inconsistent. If qRT-PCR verifies a specific transcript and transcriptome sequencing yields gene-level expression, there may be cases where expression levels are inconsistent, but expression trends are consistent.

## 4 Discussion

MO stem-base rot first appeared in 1979, researchers found MO stem-base, roots, and seeds can be infected with stem-base rot (Cheng and Xu, 1979). MO stem-base rot is caused by Fon and can lead to rot and deterioration of the stem-base. At present, the control of this disease mainly relies on chemical control, but the control effect is not ideal. It has been reported that some biocontrol bacteria can antagonize stem-base rot bacteria and induce systemic disease resistance in crops. For example, Hou et al. (2022) obtained a stain of *streptomyces sclerous* SM3-7 from the rhizosphere soil of corn, which showed good potted control against stem-base rot. Chen et al. (2022) found that the co-culture of *Trichoderma verticillium* HB20111 and *Trichoderma harzianum* TW21990 had a synergistic effect, and the control effect on stem rot at the wheat seedling stage was significantly higher than that of a single strain. However, biocontrol agents have seldom been used to control the stem-base rot of MO. In this study, we report for the first time that *Trichoderma harzianum* and *Pestalotiopsis* sp. can be used as biocontrol fungi against the stem-base rot of MO, and the control effect is better when they are used together (Figure 2).

In a common microecological environment, biocontrol bacteria compete with pathogenic bacteria for limited space and nutritional resources through rapid propagation and growth, thus limiting the growth of pathogenic bacteria, which is called competition. For example, Zheng et al. (2023) found that biocontrol bacteria *Penicillium oxalicum* QZ8 and *Trichoderma asperellum* QZ2 inhibit the growth of Fon through spatial competition. In addition to the competitive effect, biocontrol bacteria also identify plant pathogens, carry out entanglement and adsorption on pathogenic bacteria, and dissolve the mycelia of pathogenic bacteria, so as to parasitize in pathogenic bacteria and obtain the nutrients of pathogenic bacteria for growth and reproduction. For example, Harman et al. (2004) found that *Trichoderma* produces a series of enzymes to degrade the cell wall of *Fusarium*, and completes the process of hyperparasitism to *Fusarium* through recognition, contact, entrench, penetration, and parasitism. In our study, both *Trichoderma harzianum* and *Pestalotiopsis* sp. showed different degrees of resistance to the stem-base rot pathogen Fon, and parasitic on pathogenic bacteria mycelia through identification, entanglement or attachment, and finally penetrating and dissolving pathogenic bacteria mycelia (Figure 1). In recent years, there have been many reports that biocontrol bacteria can cause ISR, including those that may cause ISR in *Fusarium*. Sakineh et al. (2019) reported that the use of *S. enissocaesilis* strains IC10 and *S. rochei* strains Y28, or after the application of plant, hormones can induce ISR to Fon. Besides, some non-pathogenic fungi like *Fusarium moniliforme* can also induce ISR against Fon (Patil et al., 2011). In our study, the activities of defense-related enzymes (POD and SOD), chlorophyll content, and soluble sugar content were determined. The results showed that, compared with the control treatment, biocontrol fungi could improve the activity of defense-related enzymes, chlorophyll content, and soluble sugar content. However, the levels of 1-ketose and nystose in MO were decreased, and the levels of fructose and glucose were increased (Figure 3; Supplementary Table S4; Kashyap et al., 2021).

At present, high-throughput transcriptome sequencing technology has been widely used to study the interaction mechanism between plants and pathogens, and a large number of functional genes related to plant disease resistance have been mined, revealing the pathogenesis of pathogenic bacteria and the molecular mechanism of plant disease resistance. However, there have been no reports on the mechanism of preventing and controlling MO stem-base rot from the transcriptome level (Xu et al., 2020). In this study, we identified tens of thousands of genes that function on ISR to Fon from biocontrol fungi in MO. By treating biological control fungi alone or pretreating them before inoculation with Fon, the expression of a large number of genes in MO can be altered or modified (Figure 4; Supplementary Tables S5, S6), suggesting that biological control fungi can induce systemic resistance to Fon by altering the transcription level of MO genes. Furthermore, through our data analysis, we found that many genes are involved in regulating this regulatory process. TFs play essential roles in the immune response of plants to various stresses (Fu et al., 2019; Li et al., 2021). The transcription factors involved in stress response mainly include NAC, WRKY, bHLH, MYB, and AP2/ERF (Garg et al., 2014; Aditya and Aryadeep, 2017; Peng et al., 2021). WRKY has been shown to play an important role in plant response to pathogens. Some WRKYs play a positive regulatory role in plant defense against pathogen stress. For example, in chrysanthemums, CmWRKY15 plays a positive regulatory role in chrysanthemum resistance to stem rust by influencing the SA signaling pathway (Bi et al., 2021). In addition, some MYB factors can also enhance plant resistance to pathogens by regulating the SA pathway (Seo and Park, 2010). Besides, it has been shown that ERF proteins regulate ET/JA or SA-mediated signaling pathways in plants, resulting in moderate disease resistance responses (Liang et al., 2008). In our study, based on RNA sequencing data, we identified many TFs involved in the systemic resistance process of MO to Fon induced by biocontrol fungi, including WRKY, MYB, bZIP, ARF, and ERF, etc. (Figure 8). Among them, WRKY transcription factors are able to respond to the MAPK signaling pathway, while ERFs transcription factors are able to respond to the ethylene signaling pathway, which together regulate downstream defense-related genes.

Studies have shown that the MAPK signaling pathway plays a crucial role in promoting and protecting plant growth when plants are subjected to environmental stress (Bi and Zhou, 2017). The response of the MAPK signaling pathway to plant diseases is mainly divided into three parts, including pathogen invasion, pathogen attack, and plant hormone. After pretreatment by biocontrol bacteria, DEGs are significantly enriched in the MAPK signaling pathway. As can be seen from Supplementary Figure S6A, related DEGs in this pathway are up-regulated, including WRKY transcription factor 33(TRINITY_DN531732_c5_g3), disease-related protein 1 (PR1, TRINITY_DN536232_c0_g2), ethylene non-sensitive protein 3 (EIN3, TRINITY_DN542286_c4_g5) and alkaline endochitinase B (Chi B, TRINITY_DN526230_c1_g3, TRINITY_DN526230_c1_g2). WRKY transcription factors play an important role in plant defense against stem basal rot, and one of the important mechanisms is to regulate the downstream defense-related genes together with the MAPK signaling pathway. MAPK phosphorylates or dephosphorylates WRKY transcription factors, thereby altering the function and activity of WRKY transcription factors, which in turn regulate the expression of downstream defense-related genes. For example, in *Arabidopsis thaliana*, MPK3 and MPK6 can phosphorylate WRKY33 and increase its affinity for W-box elements, thereby promoting the expression of their target genes, such as genes involved in plant secondary metabolic pathways, such as PAD3 and CYP71A13, as well as genes involved in plant antimicrobial pathways, such as PDF1.2 and PR4. The expression of these genes enhances plant resistance to stem basal rot pathogens (Eckardt, 2011; Sheikh et al., 2016). In summary, WRKY transcription factors and the MAPK signaling pathway regulate plant resistance to stem-base rot through synergistic actions.

Hormone-regulated genes play a crucial role in plant growth and development as well as in responding to biotic stresses (Pieterse et al., 2012). Among them, SA, JA, and ethylene are known to be key phytohormones in response to biotic stresses (Lazebnik et al., 2014). In this study, this signaling pathway was activated when treated with biocontrol fungi, mainly involving hormone signaling of auxin, gibberellin, abscisic acid, ethylene, JA, and SA (Supplementary Figure S6B). In the ethylene signaling pathway, the EIN3 protein (TRINITY_DN542286_c4_g5) was up-regulated to promote the transcription of ethylene response genes. Ethylene is an important phytohormone that induces the expression of disease resistance-related genes when plants are infested with pathogenic bacteria, thereby increasing plant disease resistance. The ethylene signaling pathway has been extensively studied and some of the key enzymes and transcription factors have been identified. Ethylene signaling is mainly composed of ethylene receptors, CTR1, EIN2, EIN3/EIL1, etc., which constitute a negative feedback regulatory network to maintain the balance of ethylene signaling, they can sense ethylene signals and transmit them to downstream transcription factors such as ERFs (Zhao et al., 2021). The ERFs gene family is a class of plant-specific transcription factors that bind to ethylene-responsive elements (EREs) and regulate gene expression downstream of ethylene signaling, which can regulate biological processes such as glucose metabolism, cell wall degradation, pigment synthesis, and aroma synthesis (Zhou et al., 2022). Studies have shown that the ethylene and sugar signaling play important roles in regulating plant growth and development and in response to stress (Etzler, 1998; Shi et al., 2019). In this study, we found that the infection of Fon into the root of MO can lead to differential enrichment of glucose-related metabolic pathways. Ethylene receptor, ERFs, EIN3, ein2 et al. genes are annotated into glycosyl compound metabolic processes (GO: 1901657) and sugar-mediated signaling pathways (GO: 0010182). Therefore, the ethylene signaling may mediate the co-involvement of the sugar signaling in the immune response of MO to Fon infection. These results suggest that the ethylene signaling pathway in MO play an important role in biocontrol fungi triggering plant immunity to Fon.

Overall, the *in vitro* antagonistic results showed that *Trichoderma harzianum* and *Pestalotiopsis* sp. Inhibited Fon. Combined with the results of the greenhouse experiments, it was verified that both fungi could be used as biocontrol fungi to control MO stem-base rot, and the detection of physiological and biochemical indexes reflected that a variety of metabolic processes and functions in the plant changed after the use of the two biocontrol fungi, such as increasing the activity of antioxidant enzymes, chlorophyll content, and soluble sugars, which reflected that biocontrol fungi could promote the response of MO to Fon stress from the side. Continuing to explore the biocontrol mechanism by transcriptome sequencing, the results showed that *Trichoderma harzianum* and *Pestalotiopsis* sp. could affect the gene expression of MO and regulate the signal transduction and phytohormone pathways related to Fon resistance, such as the MAPK signaling pathway and the ethylene signaling pathway, which could improve the disease resistance and growth of MO.

## Data availability statement

The data presented in the study are deposited in the NCBI repository, accession number PRJNA1043455.

## Author contributions

ZiC: Writing – original draft, Writing – review & editing. PH: Writing – review & editing. ZL: Writing – review & editing. ZeC: Writing – review & editing. JC: Writing – review & editing. XC: Writing – review & editing. TS: Writing – review & editing. PD: Writing – review & editing.
