# Supplementary figures and images for: Biocontrol fungi induced stem-base rot disease resistance of Morinda officinalis How revealed by transcriptome analysis
Source: Front Microbiol. 2023 Dec 1;14:1257437. doi: 10.3389/fmicb.2023.1257437 (PMC10722274; doi:10.3389/fmicb.2023.1257437)

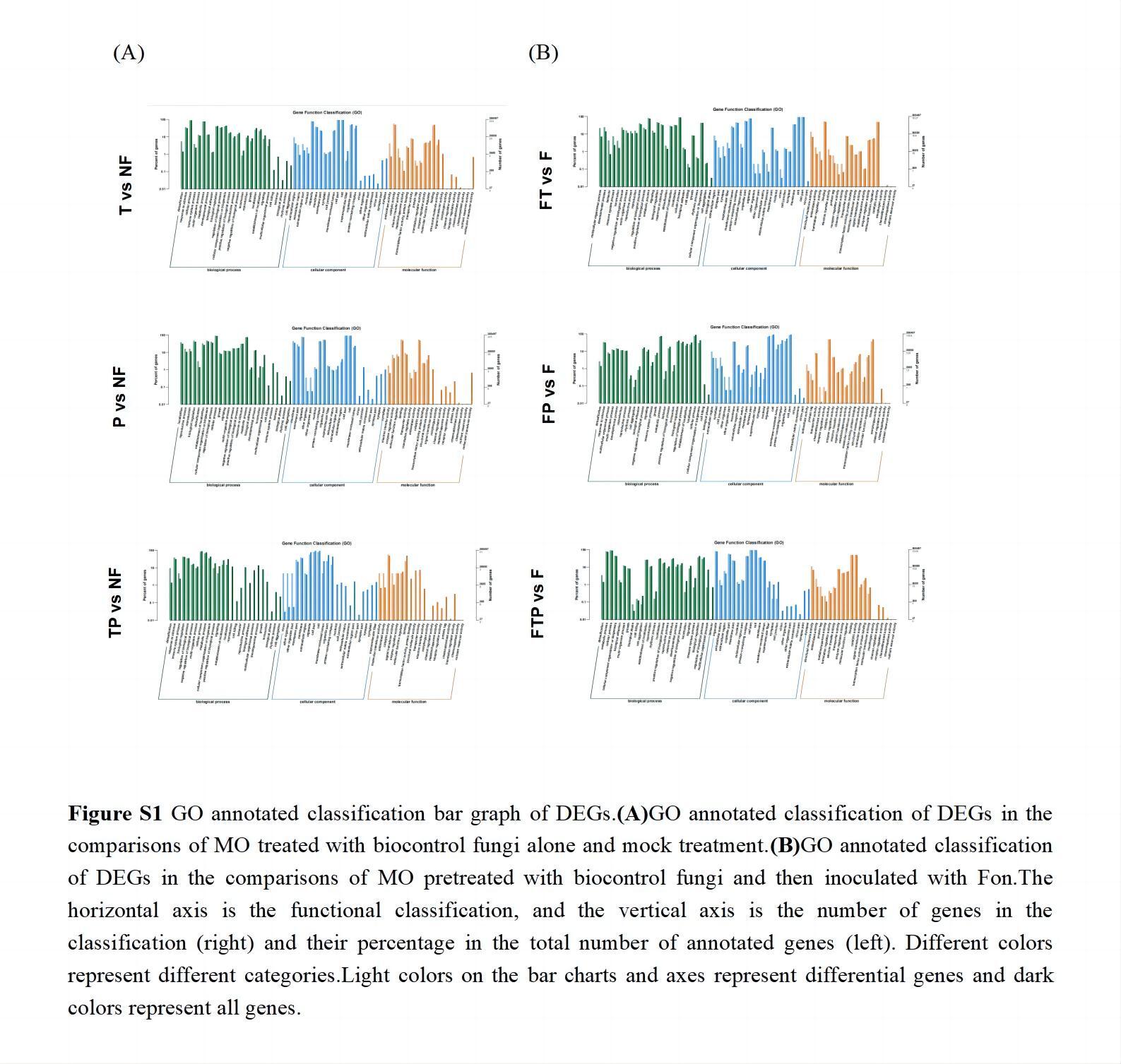

Supplement: Supplementary file 1 [file Data_Sheet_1.zip › Figure S1.JPEG]

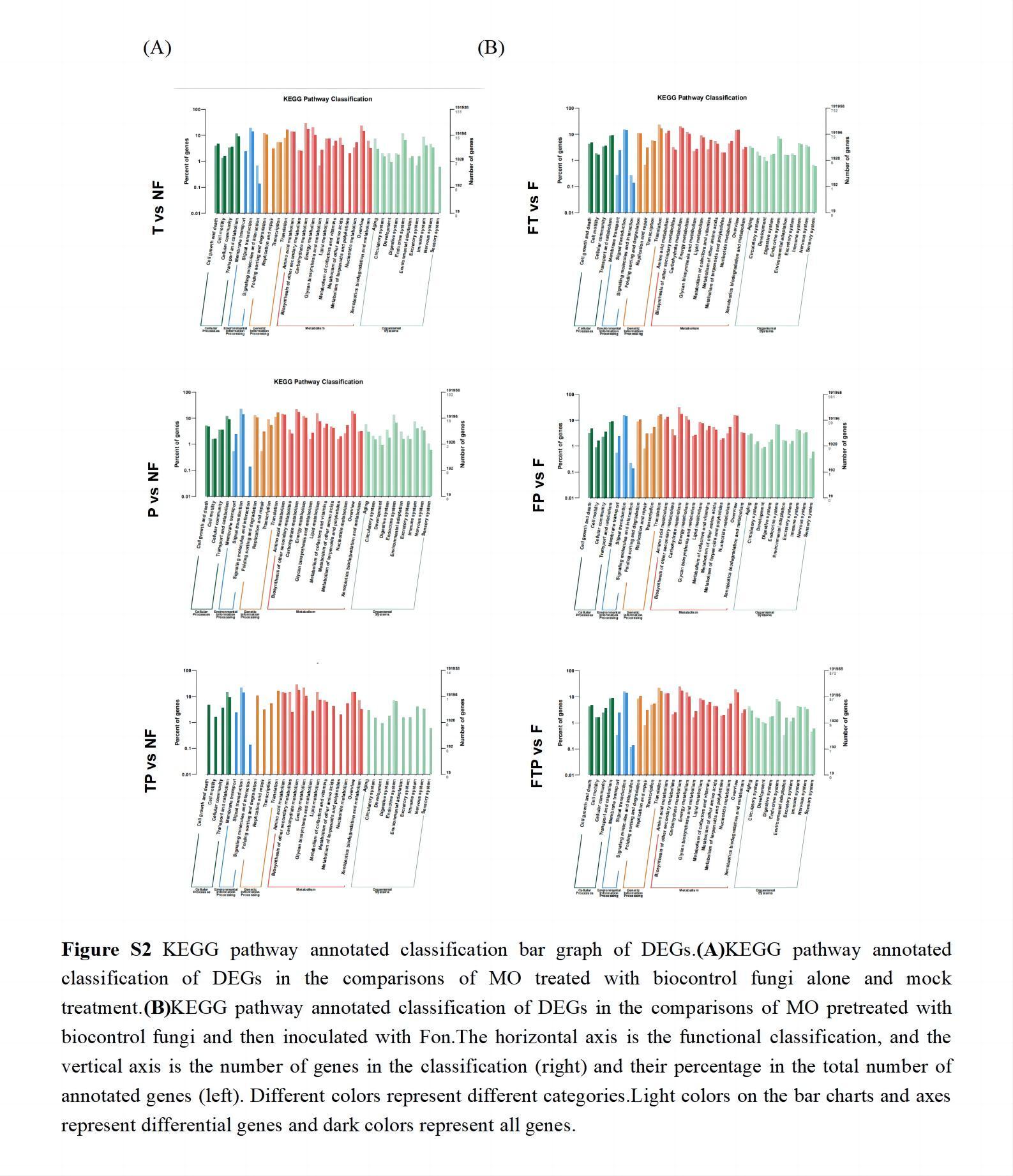

Supplement: Supplementary file 1 [file Data_Sheet_1.zip › Figure S2.JPEG]

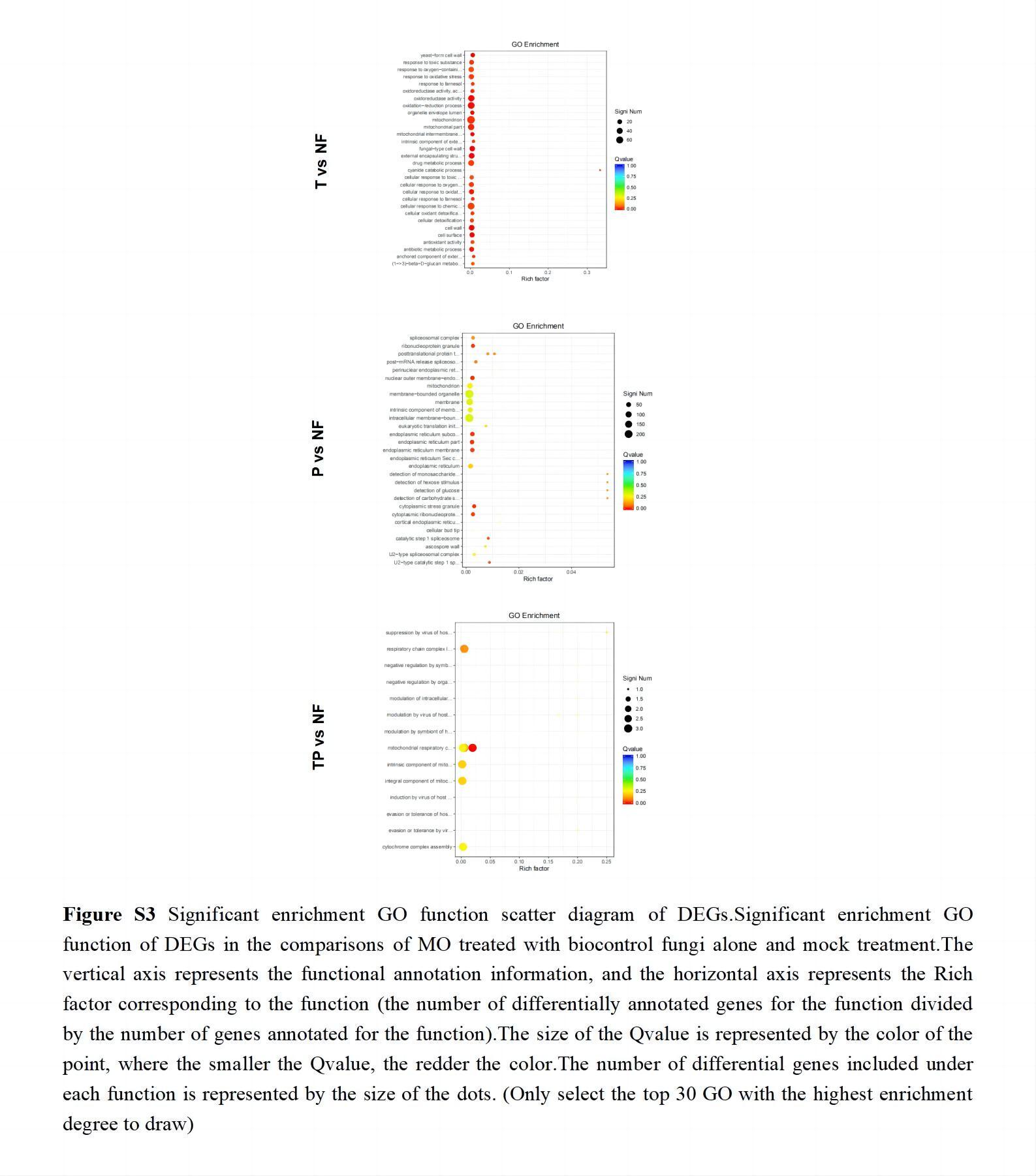

Supplement: Supplementary file 1 [file Data_Sheet_1.zip › Figure S3.JPEG]

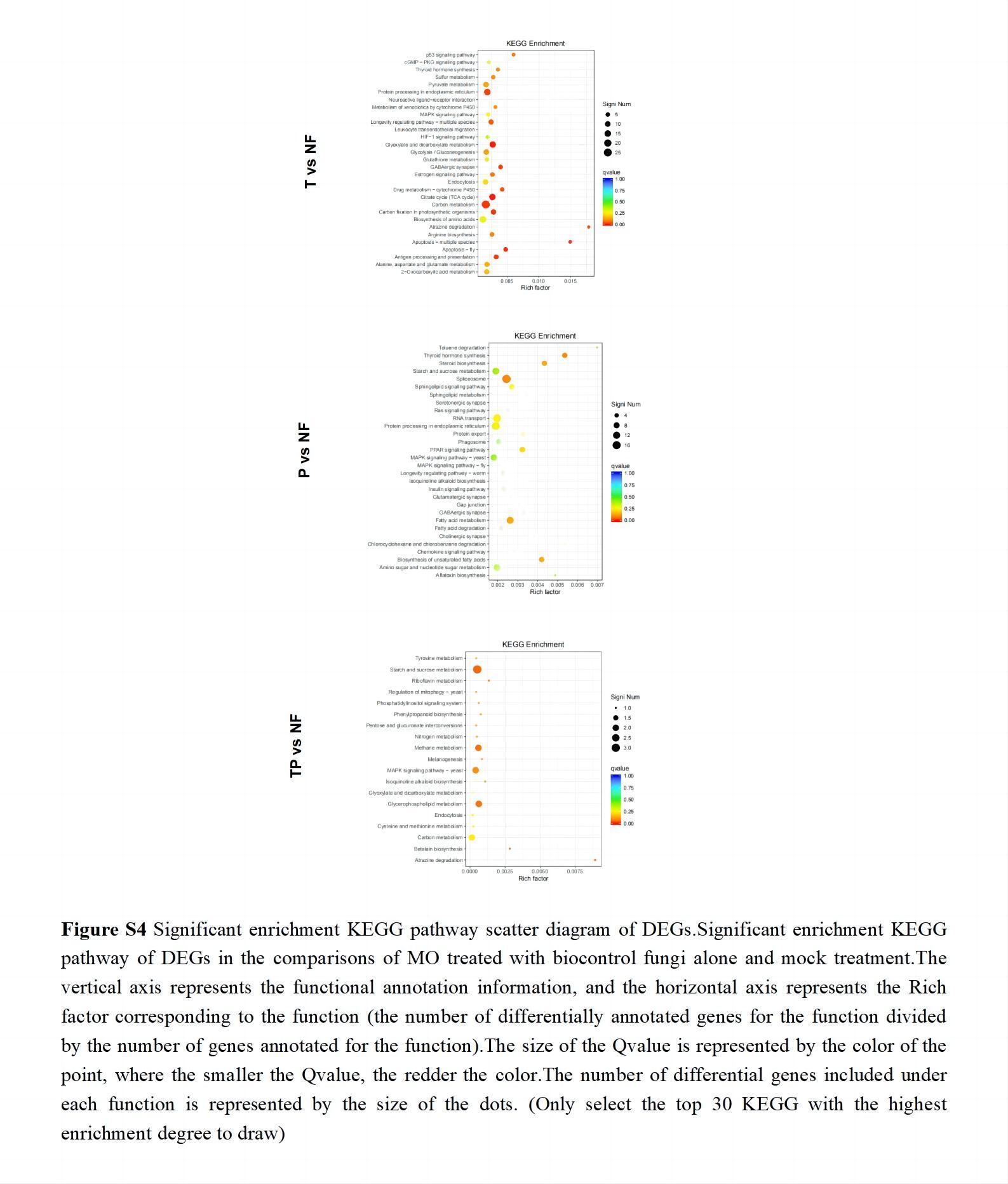

Supplement: Supplementary file 1 [file Data_Sheet_1.zip › Figure S4.JPEG]

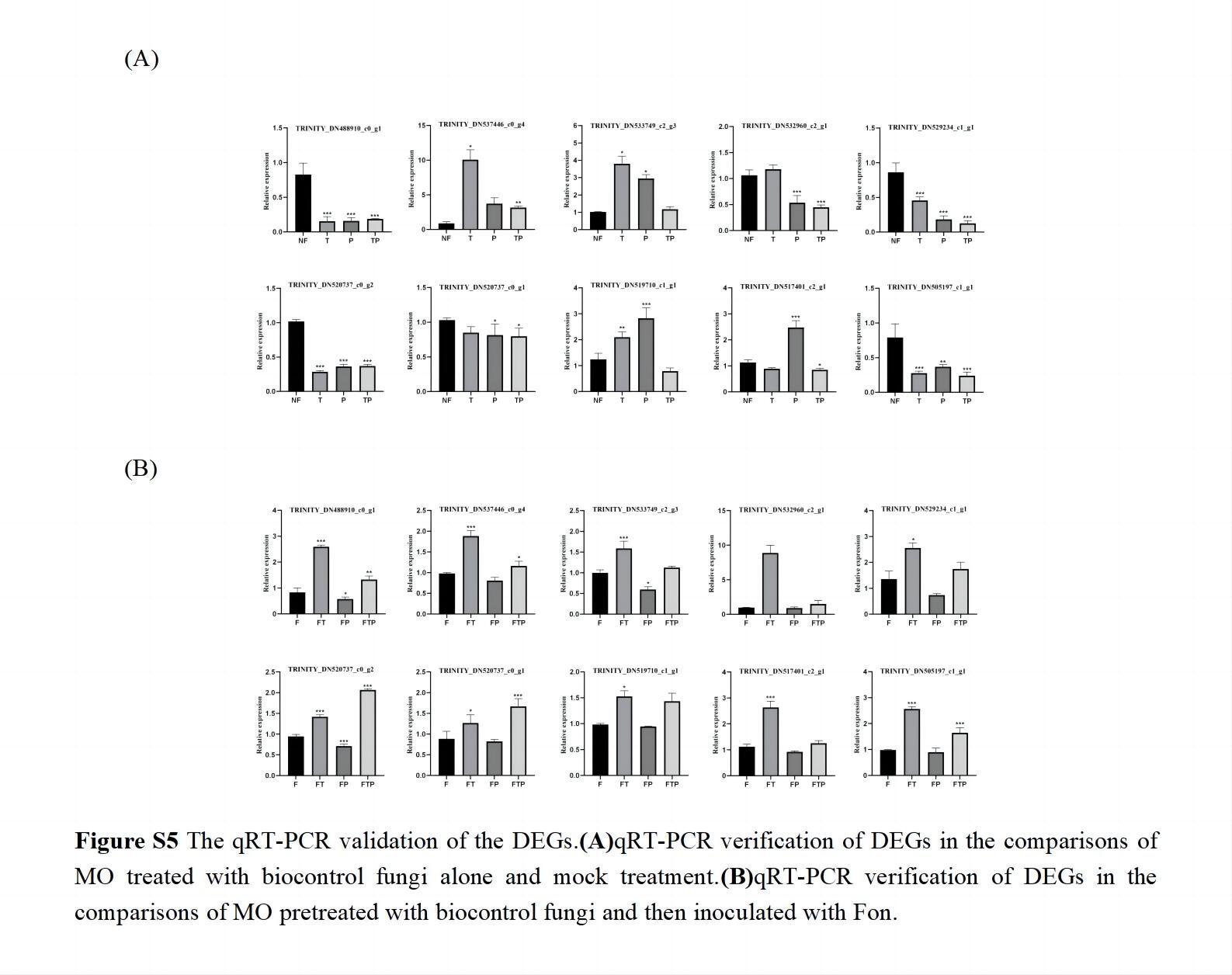

Supplement: Supplementary file 1 [file Data_Sheet_1.zip › Figure S5.JPEG]

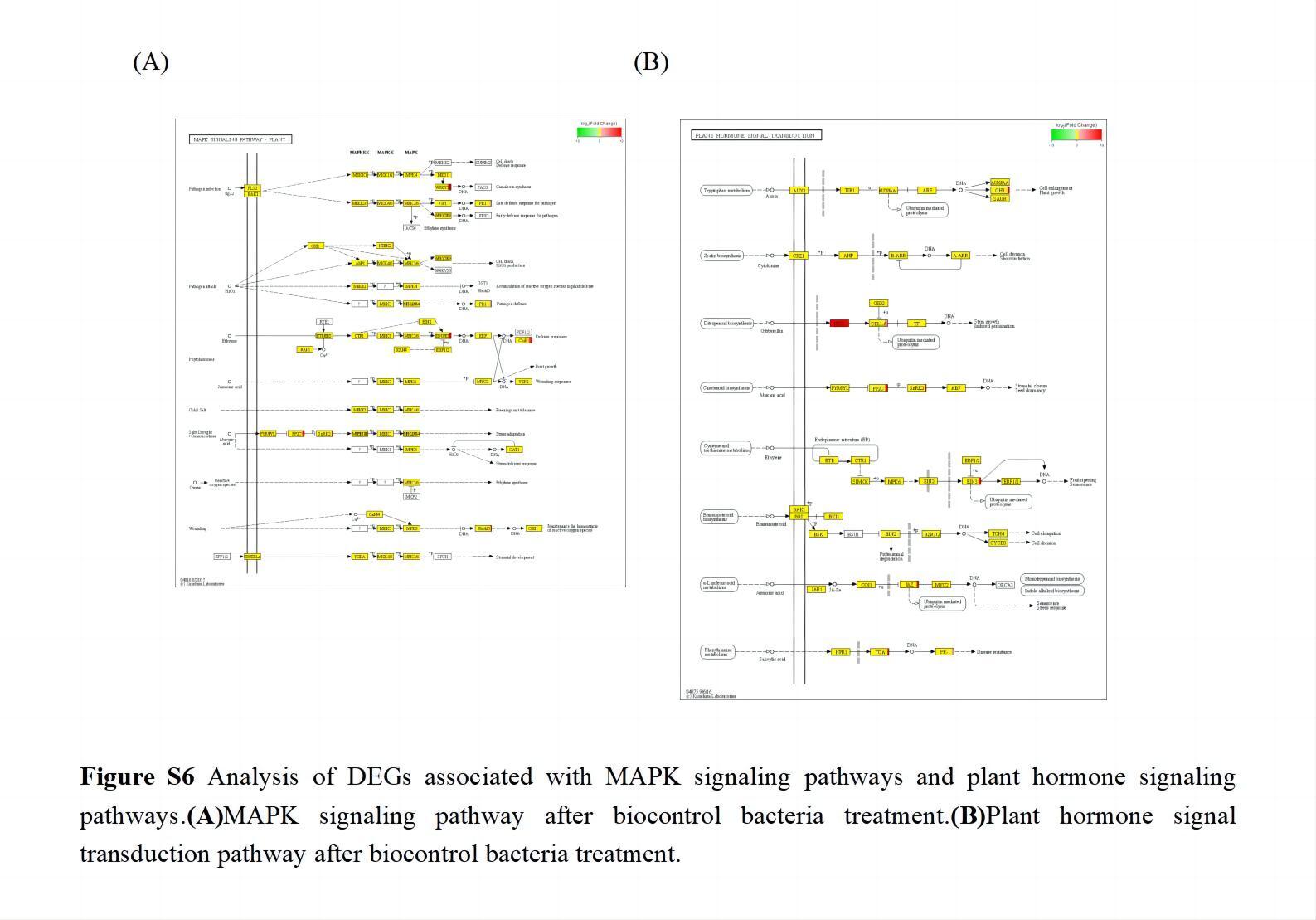

Supplement: Supplementary file 1 [file Data_Sheet_1.zip › Figure S6.JPEG]
